# Assessment of Effects of Discharged Firefighting Water on the Nemunas River Based on Biomarker Responses

**DOI:** 10.3390/toxics14010041

**Published:** 2025-12-30

**Authors:** Laura Butrimavičienė, Virginija Kalcienė, Reda Nalivaikienė, Kęstutis Arbačiauskas, Kęstutis Jokšas, Aleksandras Rybakovas

**Affiliations:** 1 State Scientific Research Institute Nature Research Centre, Akademijos Str. 2, LT-08412 Vilnius, Lithuania; reda.nalivaikiene@gamtc.lt (R.N.); kestutis.arbaciauskas@gamtc.lt (K.A.); kestutis.joksas@gamtc.lt (K.J.); 2 Institute of Biosciences, Life Sciences Center, Vilnius University, Saulėtekio Ave 7, LT-10257 Vilnius, Lithuania; virginija.kalciene@gf.vu.lt; 3 Faculty of Chemistry and Geosciences, Institute of Geosciences, Vilnius University, M. K. Čiurlionio Str. 21/27, LT-03101 Vilnius, Lithuania

**Keywords:** tire fire, *U. pictorum*, PAH and metals, aquatic toxicology, biomarkers

## Abstract

This study estimates the levels of chemical contamination and the responses of biochemical and cytogenetic biomarkers in *Unio pictorum* from the Nemunas River after a large-scale fire at a tire storage and processing warehouse (in October 2019), as well as after the subsequent discharge of partially cleaned water used for firefighting. The impact of firefighting water (FW) on the River Nemunas ecosystem was assessed. Elevated levels of trace metals (Pb, Cu, Co, Cr, Al, Zn) in *U. pictorum* mussels collected downstream from the wastewater treatment plant (WTP) discharger were measured in the first year after the accident. Genotoxic aberrations in gill cells were significantly more frequent in mussels collected downstream of the WTP discharger, along with higher frequencies of cytotoxic damage and changes in acetylcholinesterase activity. PAH metabolite concentrations, including naphthalene (Nap) and benzo(a)pyrene (B(α)P), were also elevated in haemolymph in *U. pictorum* gathered downstream from the discharger, but differences were not statistically significant. The total sum of 16 PAH concentrations in mussels collected in 2021 and 2022 was over 5 times higher than those in 2020, and the profile of accumulated metals shifted, with Ni, Cd, Cr, and Pb concentrations decreasing while Zn increased significantly. Mussel haemolymph in 2021 contained the highest levels of B(α)P-type PAH metabolites, indicating increased oxidative stress and neurotoxic impact. The results of chemical analysis and the values of genotoxic aberrations determined in gill cells of *U. pictorum* collected in 2021 and 2022 indicate an increase in PAH contamination and geno-cytotoxic impact compared to the results of 2020; these changes might be related to the gradual cancellation of COVID-19 restrictions and restoration of routine activities. The study provided an opportunity to demonstrate the unique response of a less anthropogenically stressed ecosystem to the extreme impact of contamination related to the fire on the tire recycling plant.

## 1. Introduction

On the 16th of October 2019, a large-scale fire started in the Lithuanian city of Alytus, at one of the largest tire recycling plants in the Baltic States. The fire affected an area of approximately 2000 m^2^ of a warehouse containing a large quantity (approximately 4000 t) of used and recycled tires. The combustion of tires during the fire, accompanied by the release of an additional 220 tons of particulate matter into the atmosphere, resulted in a significant increase in polycyclic aromatic hydrocarbon (PAH) and metal concentrations in the surrounding environment [1]. Moreover, during firefighting efforts, around 50,000 m^3^ of contaminated water was generated which contained over a hundred organic pollutants, as well as trace metals. The decision was made to collect and to store such firefighting water (FW) in Alytus City wastewater treatment plant (WTP), located near the River Nemunas. Subsequently, the notifications stating that the partly cleaned FW had already been started to be discharged into the Nemunas River environment were only received in spring of 2020. According to Gefenienė and co-authors (2024), research, in the FW, concentrations of ∑8 ECHA PAH (Ant, Flu, Nap, BaP, BbF, BkF, BgP and InP) were much higher than those in the Nemunas River and the WHO limits for coastal and surface waters. The study also identified the presence and increase in additional pollutants—such as metals, diesel, gasoline, alkenes, phenols, nitrogen and sulphur compounds, and others—commonly associated with tire fires, whose harmful impacts on aquatic organisms are well-known and thoroughly documented [2]. Many such PAHs were detected in the surrounding environment and in FW following the fire, including compounds that have previously been characterized as toxic, genotoxic, and carcinogenic [3], with 16 of them assigned as priority pollutants by the U.S. Environmental Protection Agency (EPA).

The screening of potentially hazardous chemicals in aquatic ecosystems is challenging because there are hundreds of thousands of toxic compounds. Moreover, many of these compounds could possibly cause a potential threat while being below the detection limit. Interactions among different components of the complex mixtures might result in changes in their impact (additive, antagonistic, synergic, etc.) on aquatic organisms and may not be accurately predicted based on previous analytical data [4,5]. For this reason, the Water Framework Directive (WFD) suggests a combination of targeted chemical analysis with effect-based methods to assess ecological impact caused by chemical contamination of European water bodies [6,7].

Despite the impossibility of predicting such incidents, it is nevertheless essential to monitor ecosystems in order to identify the threats they pose and predict future risks. According to the Lithuanian authorities, the estimated financial loss resulting from the fire is between EUR 5 million and EUR 13 million. However, the long-term impact on the affected ecosystems remains uncertain and difficult to assess.

The Nemunas River is of significant ecological importance, as evidenced by the presence of at least 4 main Natura 2000 sites along its course. These areas are of crucial importance for the maintenance of the river’s ecosystems and for the protection of biodiversity. Furthermore, the river provides a variety of ecosystem services. As stipulated by the Water Framework Directive (WFD), all EU Member States are obligated to monitor water bodies and implement measures aimed at preserving their ecological integrity.

In the present study, biological responses-based and chemical analyses were conducted on *Unionidae* family mussels collected in situ over a three-year period. Such a study offers valuable insights into the health of the target ecosystem and can be effectively used to assess the impact of contamination [8]. Numerous previous studies have highlighted the sensitivity and suitability of these organisms for assessing various biomarker responses, both *in situ* and through laboratory-based studies [9,10,11,12,13,14]. Biological, physiological, and species-specific characteristics, such as wide distribution, large populations, sedentary lifestyles, intensive filtration capacities, and contaminant accumulation rates, also contribute to the widespread use of bivalves as indicator organisms [8,11,15]. A battery of bioassays provides more precise information about the health of bioindicators. Biochemical and cytogenetic biomarkers serve as sensitive and informative warning tools for the evaluation of the impact of pollution on different types of aquatic ecosystems [7,11,12].

The total antioxidant capacity is one of the most frequently used biomarkers for assessing responses resulting from the disruption of the redox balance of an organism by a variety of stressors [16]. According to Franco-Martinez and co-authors’ research, the Ferric Reducing Antioxidant Power (FRAP) assay served as a biomarker of oxidative stress, showing higher values of FRAP in the digestive gland and hemolymph of hypoxic *Mytilus galloprovincialis* in comparison to the control group. Fluorescence assays can be used to detect PAH metabolites in various vertebrate and invertebrate fluids (bile, urine, and haemolymph) as biochemical biomarkers in the assessment of environmental contamination by petrogenic and pyrogenic PAHs [17,18,19]. The fixed wavelength fluorescence (FF) method was proposed as a more cost-effective alternative to the High-Performance Liquid Chromatography with Fluorescence Detection (HPLC-F) method for analysis of PAHs in environmental pollution assessment [19].

Acetylcholinesterase (AChE) activity is frequently employed as a biomarker for evaluating neurotoxic effects in organisms exposed to various pollutants [20]. AChE is an enzyme that is predominantly located at cholinergic synapses, especially in muscles and nerves. It immediately breaks down or hydrolyses the neurotransmitter acetylcholine. Inhibition of this enzyme leads to the blocking of nerve impulse transmission [21,22].

Increased frequencies of geno- (nuclear buds (NB), micronuclei (MN), and nucleoplasmic bridges (NPBs)) and cytotoxicity (binucleated cells (BN) and fragmented-apoptotic (FA)) parameters serve as informative biomarkers of contamination with genotoxic and cytotoxic agents in various ecosystems and various organisms [23,24,25]. Genotoxic compounds can interact with DNA and enzymatic complexes and cause the formation of various genetic disturbances, influence replication and transcription processes, interfere with the normal functioning of the cell, and, in most cases, induce mechanisms that lead to cell death (cytotoxicity) [26]. If the reparation system fails, the non-lethal genotoxic impact causes accumulation of DNA damage, which eventually increases the risk of developmental and degenerative diseases, reproductive disorders, and abnormal physiological responses and, at the same time, might lead to genome instability and formation of cancer [7]. Extensively, the genotoxic effects could lead to a reduction in reproductive potential, affect biodiversity, and ultimately lead to changes in the entire ecosystem [11].

The aim of the study was to assess the impact of FW discharged from Alytus City WTP on the Nemunas River ecosystem’s health. Four study sites along the Alytus WTP outlet in the Nemunas River were investigated: one upstream and three downstream. The environmental status of the Nemunas River was assessed over a three-year period using the biological indicator *U. pictorum*. Evaluation of “stress-based” biological responses (AChE, PAH metabolites, antioxidant capacity, and environmental geno- and cytotoxicity) and bioaccumulation of contaminants (trace metals and PAHs) was performed.

## 2. Materials and Methods

### 2.1. Study Sites and Collection of Mussels

**Figure 1:**
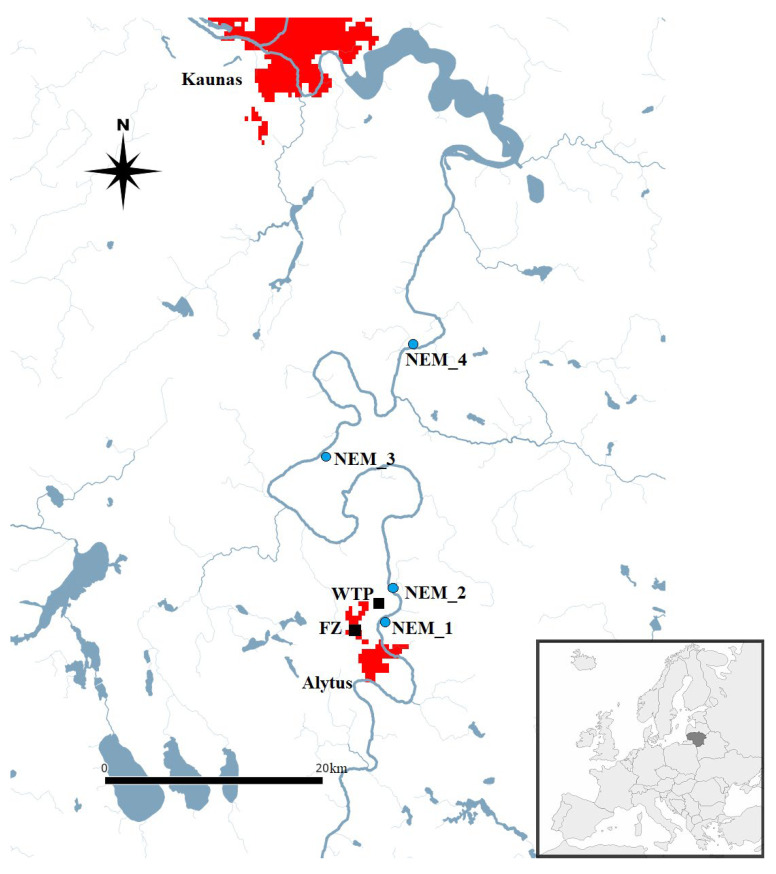
Map indicating the location of fire zone (FZ), Alytus City wastewater treatment plant (WTP), and sampling sites (NEM_1, NEM_2, NEM_3, and NEM_4) in the River Nemunas marked in blue, with the urban area marked in red. The subfigure marks the location of Lithuania (dark grey) in Europe (light grey).

*U. pictorum* mussels were collected in June 2020, 2021, and 2022 at four Nemunas River sites (Figure 1). The abbreviations of stations, sampling geographical coordinates, sampling dates, and short descriptions of stations are presented in Table 1. At each study site, 25 specimens of *U. pictorum* with a similar body size (overall mean of length 77.19 ± 0.57 mm; weight 34.79 ± 0.99 g) and at the same age (the number of growth rings on shells varied from 6 to 8 years [27]) were hand-collected in wadable depths (~0.5–1.2 m). Mussels were placed in plastic tanks filled with aerated water from the sampling location and transported to the laboratory at the State Scientific Research Institute Nature Research Centre (NRC). Biochemical and cytogenetic biomarkers were analysed using 10 organisms from one study station, while accumulation of trace metals and PAHs was assessed in 15 specimens.

### 2.2. Chemicals

All chemicals used in this study were of analytical grade or higher and acquired from Carl Roth (Karlsruhe, Germany), Sigma-Aldrich (Darmstadt, Germany), and BioRad (Hercules, CA, USA).

### 2.3. Methods

#### 2.3.1. PAHs Analysis in Mussels

For mussel soft tissue samples, a combined method based on procedures described previously by Kalachova et al. [28] and Guillen et al. [29] was used: approximately 2 g of homogenized sample was saponified with 200 mL of methanolic KOH in the ultrasonic bath at 60 °C for 30 min. Samples were then filtered through glass wool and extracted with 100 mL of dichloromethane (DCM; HPLC grade, Sigma-Aldrich, Darmstadt, Germany) in an ultrasonic bath for 30 min. After the transfer of the DCM layer to a rotary evaporator flask, the extraction procedure was repeated 2 times more. The extracts were combined and concentrated to approximately 2 mL under vacuum. Extracts were cleaned with silica SPE cartridges, and DCM was used for the elution of the analytes. The concentration of purified extracts was performed using a gentle steam of N_2_, and the analytes were reconstituted in 300 µL of acetonitrile.

The analysis and compounds identification was performed by the methodology described in previously published studies [1,30]. Concentrations of each PAH (16 PAHs: naphthalene (Nap), acenaphthylene (Acy), acenaphthene (Ace), fluorene (Fl), phenanthrene (Phe), anthracene (Ant), fluoranthene (Flu), pyrene (Pyr), benzo(a)anthracene (BaA), chrysene (Chr), benzo(b)fluoranthene (B(β)F), benzo(k)fluoranthene (B(κ)F), benzo(a)pyrene (B(α)P), indeno(1,2,3-cd)pyrene (InP), dibenzo(ah)anthracene (DbA), and benzo(ghi)perylene (BgP)) were determined using the external standard method, with the range of the calibration curve from 0.01 to 1 μg/mL.

The procedural blanks and replicate samples (n = 3) were used for quality control. Relative standard deviation (RSD) was less than 10%, and no traces of the analytes of interest were observed in the chromatograms of the blank samples. Procedural blanks were analysed periodically, with one blank included for every batch of 10 samples. The efficiency of recovery of the procedure was determined by analysing matrix samples spiked with a known amount of the PAH Mixture (EPA Method 8310, Restek, Bellefonte, PA, USA). PAH recoveries varied between 75% and 112%. The lowest recoveries were for Ace and for Nap, with 75% and 81%, respectively, whereas the highest recoveries were obtained for higher molecular weight compounds, such as InP—104% and BgP—112%. The rest recoveries of the analytes ranged from 88 to 97%. Five sets of analytical blanks were used for the determination of the limit of quantification (LOQ) and the limit of detection (LOD). The LOQ and LOD of individual PAH were calculated from 3 and 10 multiples of the standard deviation (SD), respectively, of the mean of signal-to-noise ratio (S/N) for 10 measurements, in comparison to the baseline noise close to the peak of each PAH blank, respectively. LOQ varied from 0.05 to 0.70, while LOD ranged from 0.015 to 0.2.

#### 2.3.2. Metal Analysis in Mussels

Determination of metals (Pb, Ni, Cu, Co, Cr, Cd, Al, and Zn) in soft tissues of *U. pictorum* was performed with an inductively coupled argon plasma spectrometer PerkinElmer Optima 1000 DV ICP-OES (PerkinElmer, Waltham, MA, USA), following a modified procedure by Sen et al. [31] and described in detail in the study of Raudonytė-Svirbutavičienė et al. [1]. The analytical procedure precision, expressed as a standard deviation, was better than 10%. The accuracy was within 15% of the certified values for Cr and better than 10% for the other elements. The precision, expressed as RSD, was always better than 7% for the measured elements. All concentrations were calculated on a dry weight basis. Limit of quantification (LOQ) ranged from 0.02 to 0.1 mg kg^−1^ d.w., while Limit of detection (LOD) ranged from 0.006 to 0.03 mg kg^−1^ d.w.

#### 2.3.3. Preparation for Biochemical Biomarkers Analysis, Evaluation of PAH Metabolites

*Unio pictorum* for the determination of PAH metabolites, FRAP, and AChE were prepared within four hours after the transportation to NRC. Haemolymph was collected using a 1 mL syringe (B. Braun Melsungen AG, Melsungen, Germany) with a 25G needle (B. Braun Melsungen AG, Melsungen, Germany) inserted into the adductor muscle. Collected haemolymph was placed in a 1.5 mL centrifuge tube and centrifuged at 4 °C for 5 min at 3000 RPM using a centrifuge Eppendorf 5424R, (Eppendorf, Hamburg, Germany).The obtained biomaterial was stored at −80 °C in a freezer NU-9483E (NuAire, Plymouth, MN, USA) until the biochemical assays were carried out. All fluorescence and absorbance measurements for biochemical biomarker analysis were performed using a multi-mode microplate reader Tecan Infinite M200 (Tecan Group Ltd., Männedorf, Switzerland).

The method of FF was used for the assessment of PAH metabolites according to the methodology described earlier [17]. Before the measurement, haemolymph was diluted 1:100 with 50% ethanol in 96-well white microplates. Fluorescence levels of naphthalene- (FF2), and benzo(a)pyrene—(FF5) type metabolites in diluted haemolymph were measured at excitation/emission wavelength pairs of 290/335 and 380/430, respectively. PAH metabolites in each mussel were assessed in triplicate. Solvent (50% ethanol) background fluorescence was subtracted from haemolymph fluorescence. Levels of analysed PAHs are presented in relative fluorescence units.

#### 2.3.4. Assessment of Antioxidant Capacity

Antioxidant capacity in undiluted haemolymph of *U. pictorum* was assessed using Ferric Reducing Antioxidant Power (FRAP) reagent (FRAP reagent prepared in a 10:1:1 ratio by mixing 300 mM CH_3_COONa·3H_2_O buffer (pH 3.6), 10 mM 2,4,6-tri(2-pyridyl)-1,3,5-triazine 40 mM HCl solution and 20 mM FeCl_3_ solution), and FeSO_4_·7H_2_O standard as described earlier [32]. The absorbance of the reaction product after 10 min was measured in microplates at 593 nm wavelength. FRAP in each mussel was assessed in triplicate. Results were expressed as concentrations of Fe^2+^ in µM.

#### 2.3.5. Measurement of AChE Activity

Acetylcholinesterase activity in haemolymph was determined using a modified method of Ellman [33] adapted to microplates. Samples and buffer solutions (50 μL) were incubated for 5 min in microplates at 20 °C with 270 μM 5,5-dithiobis(2-nitrobenzoic acid) in 50 mM phosphate buffer, pH 7.4. Absorbance measurement at 412 nm was started after adding 3 mM acetylthiocholine iodide substrate (ACTI) and terminated after 5 min. Total protein concentrations were determined according to the Bradford method using bovine albumin as the standard [34]. AChE activity was expressed as nM of hydrolyzed substrate (ACTI) min^−1^ mg^−1^ protein using an extinction coefficient value of 1.36 × 10^4^ M^−1^ cm^−1^ for 5-thio-2-nitrobenzoic acid [35].

#### 2.3.6. Determination of Environmental Geno- and Cytotoxicity in *U. pictorum* Gill Cells

Slide preparation procedures and criteria of genotoxicity (MN, NB and NPBs) and cytotoxicity (FA and BN cells) assessment were elaborated, standardized and described during previous studies [24,36,37]. Frequencies of studied aberrations were expressed as numbers of aberrations per 1000 gill cells scored (‰). Mean values of assessed cytogenetic lesions (±SE) were calculated for mussels from each study site. For integrated genotoxicity (∑GT), we measured the summed frequencies of NB, MN and NPBs, as well as using the summed frequencies of FA and BN for integrated cytotoxicity (∑CYT).

### 2.4. Statistical Analysis

The comparison of biomarker responses in mussels from different sampling sites during respective sampling times was conducted using an open-source statistical data analysis program (PAST v4.0 and R). Data normality and variance homogeneity were checked using the Kolmogorov–Smirnov and Levene’s tests (data was homogeneous but not normally distributed), respectively. Differences between upstream (NEM_1) and downstream stations (NEM_2, NEM_3, and NEM_4) were assessed using a planned Mann–Whitney U test; for comparison, for those stations, the level of significance was established at *p* < 0.05. In graphics, the mean values are presented with standard errors (SE). Annual comparisons were performed on square-root–transformed data to meet normality and homogeneity of variance using two-way main-effect ANOVAs followed by post hoc Fisher LSD tests (Statistica 12 software package, StatSoft, Inc. Tulsa, OK, USA).

Principal Component Analysis (PCA) was applied to explore patterns in both accumulated chemicals and biological response variables. The dataset included 15 environmental predictors (Pb, Ni, Cu, Co, Cr, Cd, Al, Zn, Nap, Acy, Ace, Fl, Phe, Ant, H_PAHs (summed concentrations with high molecular weight PAHs, 4–6 rings)) and six biological response variables (∑GT, ∑CYT, AChE, FRAP, FF2, FF5). Prior to PCA, all continuous variables were log-transformed to improve normality and standardized to unit variance (centred and scaled). Categorical variables, year and site, were treated as factors for visualization purposes. PCA was conducted using the prcomp function in R (version 4.5.2, R Core Team, 2025, Vienna, Austria) [38]. A biplot was generated using the fviz_pca_biplot function from the factoextra package [39].

## 3. Results

### 3.1. Chemical Analysis in U. pictorum

**Figure 2:**
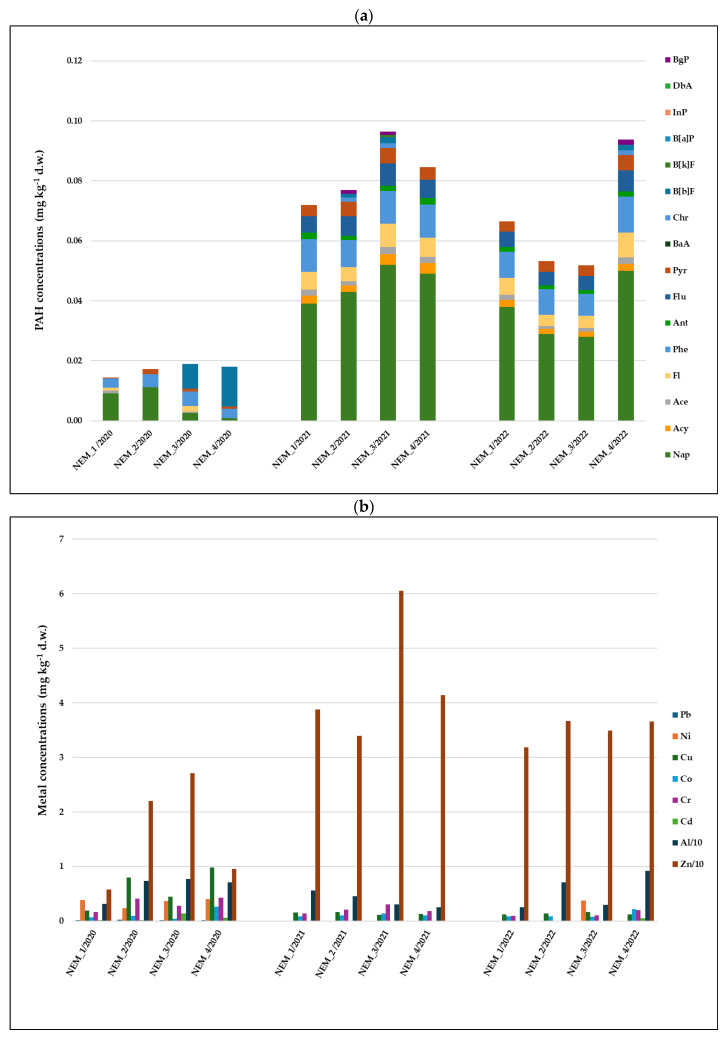
Concentrations of (**a**) PAHs (abbreviation of each individual hydrocarbon is presented in the “PAHs analysis in mussels” Section 2.3.1) and (**b**) metals in soft tissues of *Unio pictorum* from the Nemunas River. Note: Zn and Al concentrations are divided by 10 for visualization. For site location and description, see Figure 1 and Table 1.

In 2020, relatively low values of PAHs in mussels’ tissues were detected at all study stations. Total summed concentrations (16 compounds) ranged from 0.014 mg kg^−1^ d.w. at the uppermost NEM_1 station, up to 0.018 mg kg^−1^ d.w. at the NEM_4 site (Figure 2a). Mussels collected at the stations located below the point of the Alytus WTP discharger accumulated higher concentrations of Nap, Phe, and Pyr at the NEM_2 station and B[b]f at the NEM_4 station. In 2021, accumulated PAH concentrations significantly increased in all study stations: in NEM_1, total PAHs were up to 0.079 mg kg^−1^ d.w., while at NEM_3, they were −0.096 mg kg^−1^ d.w. Such values were more than 5 times higher than those assessed a year earlier. The major changes were in the concentrations of lower-molecular-weight PAHs of pyrogenic origin, although changes were also observed for petrogenic Flu and Pyr. Relatively high PAH values were also observed in mussel tissues in 2022 in the NEM_4 station, up to 0.094 mg kg^−1^ d.w, though in all other stations, those values slightly decreased compared to those assessed in 2021, including not only petrogenic but also pyrogenic compounds.

In 2020, in soft tissues of *U. pictorum*, which were collected in station NEM_1 (located upstream of the discharge point of water used for firefighting), the lowest concentrations of all studied metals, except Ni, were found (Figure 2b). Meanwhile, in the tissues of mussels from NEM_2 station, concentrations of Pb, Cr, Cd, and Al were more than 2 times higher than those at NEM_1, concentrations of Co differed by 1.3 times, while Zn and Cu were higher by more than 3.8 and 4.2 times, respectively. Mussels of the most downstream NEM_4 station contained higher concentrations of Ni, Cu, Co, Cr, Cd, and Al, while concentrations of Pb and Zn were lower compared to NEM_2. In 2021, the amounts and composition of metals accumulated in the mussels’ tissues changed: concentrations of trace metals such as Pb, Ni, and Cd were below the detection limits and concentrations of Cr and Cu were also lower in all study stations (and ranged from 0.14 (NEM_1) to 0.3 (NEM_3) mg kg^−1^ d.w. and from 0.11 kg^−1^ d.w. (NEM_3) to 0.17 kg^−1^ d.w. (NEM_2), respectively), while on the contrary, concentrations of Al and especially Zn were higher than those assessed in 2020 (accordingly up to 5.60 kg^−1^ d.w. (NEM_1) and 60.50 mg kg^−1^ d.w. (NEM_3)). In 2022, the pattern of contamination remains similar to the one determined in 2021, as concentrations of Pb, Ni, and Cd were below the detection limits, except for Ni (0.37 mg kg^−1^ d.w. at NEM_3) and Cd (0.05 mg kg^−1^ d.w. at NEM_4), and relatively high concentrations of Al (up to 9.18 mg kg^−1^ d.w. at NEM_4) and Zn (up to 36.70 mg kg^−1^ d.w. at NEM_2) were found compared to the values assessed in 2020. Compared to the other study sites, the lowest concentrations of all trace metals, except Cr, were again detected in 2022 in mussels from the uppermost station, NEM_1 (Figure 2b).

### 3.2. Biomarker Responses

#### 3.2.1. Antioxidant Capacity and PAHs Metabolites

**Figure 3:**
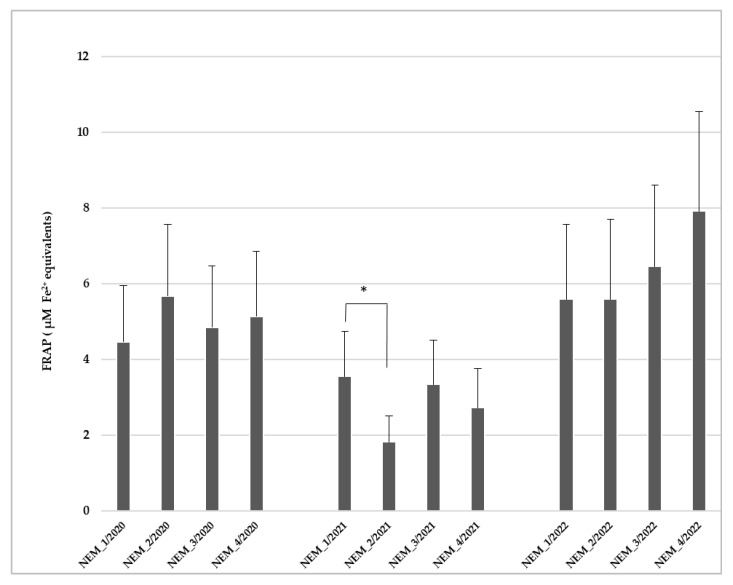
Ferric Reducing Antioxidant Power (FRAP) values expressed as µM Fe(II) in the haemolymph of *Unio pictorum* (mean ± standard error (SE), n = 10). Asterisks (*) indicate significant differences at *p* levels < 0.05 between the study groups.

A slight increase in FRAP value (5.67 ± 0.79 µM Fe^2+^ equivalents) was observed in *U. pictorum* specimens collected at station NEM_2 below the Alytus WTP outlet in 2020. Low values in 2021 were noticed in all stations, and an increase was seen in 2022 (Figure 3). The lowest FRAP value (1.81 ± 0.42 µM Fe^2+^ equivalents) was observed in mussels collected in the study site NEM_2 in 2021. A statistically significant difference between FRAP values was found in mussels collected in station NEM_2 and in specimens collected in NEM_1 in 2021 (Mann–Whitney test: *p* = 0.008).

Two-way main effect ANOVAs following post hoc Fisher LSD tests showed that FRAP values measured at 2021 were lower than those measured in 2020 and 2022 (two-way ANOVA: F_2, 94_ = 16.945, *p* = 0.000001; post hoc Fisher LSD test *p* = 0.0001 and *p* = 0, accordingly).

**Figure 4:**
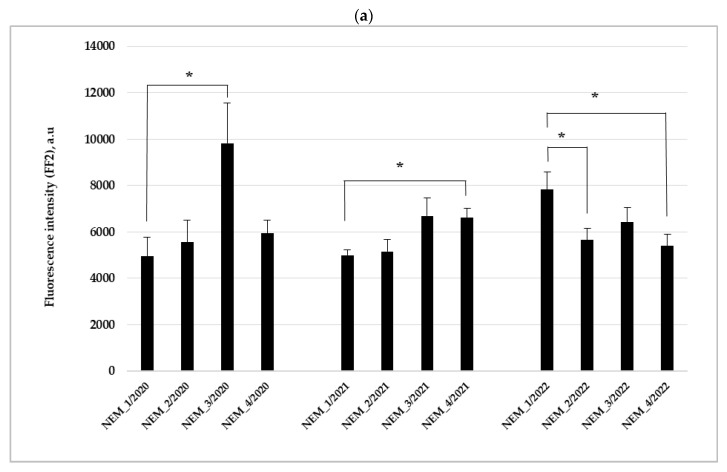

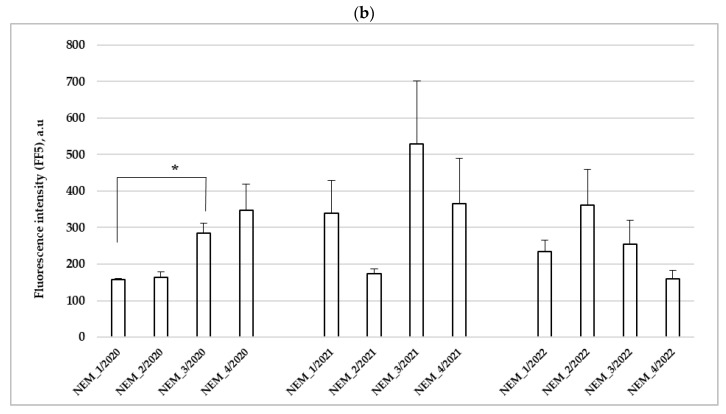
The result of fixed wavelength fluorescence analysis used for detection of the levels of (**a**) naphthalene (FF2) and (**b**) benzo(a)pyrene (FF5) type PAH metabolites in haemolymph of *Unio pictorum* (mean ± SE, n = 10). Asterisks (*) indicate significant differences at *p* levels < 0.05 between the study groups.

In 2020, the highest levels of naphthalene-type PAH metabolites were found in the haemolymph of mussels collected in the NEM_3 site (9823.23 ± 1723.67 a.u.) and were significantly different from the levels in mussels collected in NEM_1 during the same study year (*p* = 0.030) (Figure 4a). In 2021, the lowest levels of naphthalene (two benzene rings) type PAH metabolites (4988.56 ± 230.71 a.u.) were observed in mussels collected from the NEM_1 site. This level was significantly different from that recorded in mussels collected from the NEM_4 site in 2021 (*p* = 0.013). In 2022, the significantly higher levels of naphthalene-type PAH metabolites (7825.31 ± 762.60 a.u.) were recorded in mussels collected in NEM_1 station in comparison to NEM_2 (*p* = 0.034) and NEM_4 sites (*p* = 0.024).

A post hoc LSD test showed that the year effects for naphthalene-type PAH metabolites were non-significant. Only site differences were significant: station NEM_3 differed from NEM_1 (*p* = 0.024), from NEM_2 (*p* = 0.003), and from NEM_4 (*p* = 0.043). The site effect was statistically significant and was also demonstrated by the performed two-way ANOVA: F_3, 99_ = 3.374, *p* = 0.021.

In 2020 and 2021, the highest benzo(α)pyrene type PAH metabolites values were determined in *U. pictorum* collected at the stations NEM_3 and NEM_4, far from the Alytus WTP, and the differences assessed between the values determined in the station NEM_1/2020 and NEM_3/2020 were significant (Mann–Whitney test: *p* = 0.011) (Figure 4b). The first-year study revealed an increasing trend in PAH metabolite levels assessed in mussels collected downstream of the Alytus WTP discharger point. The highest levels of benzo(α)pyrene type PAH metabolites (529.5 ± 151.21 a.u.) were found in mussels collected in NEM_3 site in 2021. Two-way main effect ANOVAs following post hoc Fisher LSD tests did not show significant differences between years and study sites.

#### 3.2.2. Acetylcholinesterase Activity

**Figure 5:**
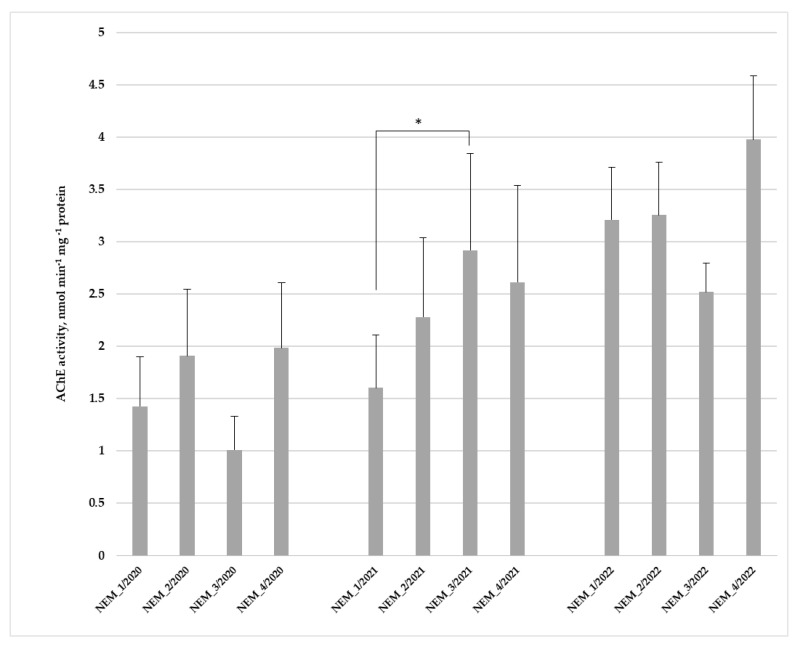
Acetylcholinesterase (AChE) activity in haemolymph of *Unio pictorum* (mean ± SE, n = 10). Asterisks (*) indicate significant differences at *p* levels < 0.05 between the study groups.

Increased AChE activity was observed in mussels collected below the outlet of Alytus WTP at station NEM_2 in 2020 (Figure 5). Also, the absolute lowest value during the first research year (2020) was estimated in *U. pictorum* from station NEM_3 (1.01 ± 0.26 nmol min^−1^ mg^−1^ protein). In the second research year (2021), a statistically significant increase in AChE activity was detected in the haemolymph of mussels collected from the NEM_3 site, in comparison to the NEM_1 site (Mann–Whitney test: *p* = 0.017). Furthermore, an increase in AChE activity tendency was determined in specimens from station NEM_1 during a monitoring period. An enhancement in enzyme activity was recorded in the third research year (2022) when compared to biomarker responses in 2020 and 2021.

Two-way main effect ANOVAs following post hoc Fisher LSD tests showed that for AChE activity only the year effects were significant for all study periods. Two-way ANOVA: F_2, 103_ = 15.646, *p* = 0.000001; post hoc Fisher LSD test: *p* = 0.001 (2020), *p* = 0.022 (2021) and *p* = 0 (2022).

#### 3.2.3. Environmental Geno- and Cytotoxicity

**Figure 6:**
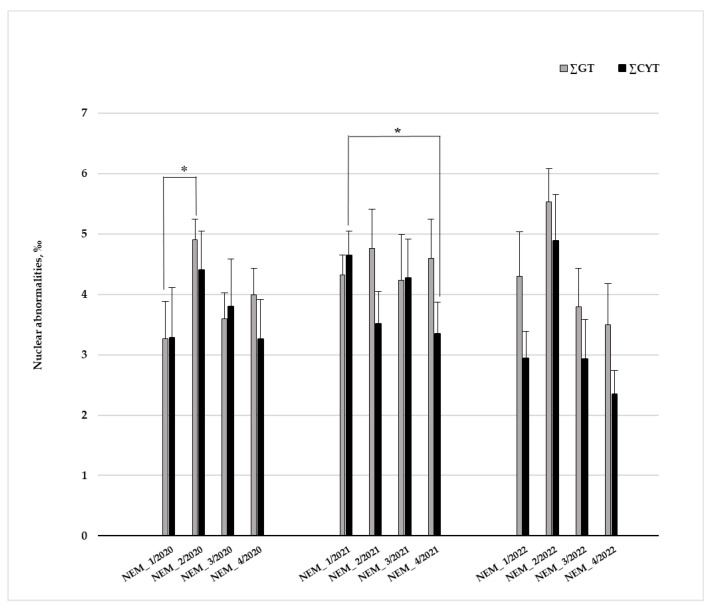
Nemunas River environmental total genotoxicity (∑GT) and total cytotoxicity (∑CYT) assessed in gill cells of *Unio pictorum* (mean ± SE, n = 10). Significant differences between the biomarkers assessed at the different study stations are marked with an asterisk (*), the level of significance *p* ≤ 0.05 (Mann–Whitney test).

In the first year of the study (2020), the highest ∑GT value—4.9‰—was assessed in mussels collected at station NEM_2 (Figure 6). The ∑GT value observed in *U. pictorum* from this site was 1.5-fold higher than the one determined in mussels from the station NEM_1 (∑GT—3.269‰); differences between the stations were significant (*p* = 0.041). The total genotoxicity value determined in mussels from NEM_1 in 2020 was the lowest of all assessed in the current study. In the following years, the highest total genotoxicity values were also estimated in mussels collected at the station NEM_2—4.76‰ (in 2021) and 5.53‰ (in 2022). The decline in ∑GT values at the NEM_3 station, in comparison to NEM_2, was observed during all survey years.

In 2021, ∑GT and ∑CYT levels in mussels of all stations studied were higher compared to the previous year. This year, ∑GT values at the NEM_1 station were higher than the ones determined in 2020, while despite the increase, total cytotoxicity differences between years were not significant. The highest value of ∑CYT (4.65‰) was determined in *U. pictorum* from the NEM_1 station, which significantly differs from the level of cytotoxic aberrations assessed at station NEM_4 (*p* = 0.024). The 2022 year of survey showed that at station NEM_1, the ∑GT value remains similar (∑GT = 4.30‰) to that assessed in 2021. Total genotoxicity and cytotoxicity values assessed in 2022 in mussels from the station NEM_2 were equal to 5.53‰ and 4.89‰, respectively, and were the highest measured over the three years of study. In mussels from stations NEM_3 and NEM_4, estimated values of ∑GT (3.79‰ and 3.5‰, respectively) and ∑CYT (2.93‰ and 2.35‰, respectively) were much lower than those at the NEM_2 station (Figure 6).

For ∑GT, year effects were non-significant (two-way ANOVA: F_2, 114_ = 0.66, *p* = 0.52), while study sites revealed significant effects (F_3, 114_ = 2.89, *p* = 0.038). Values measured at the NEM_2 were higher than those measured at NEM_1 (post hoc Fisher LSD test *p* = 0.021), while stations NEM_3 and NEM_4 (*p* < 0.031). post hoc Fisher LSD tests showed a similar tendency for ∑CYT data, where higher values were assessed at NEM_2 (expect, 2021) in 2020 and 2022, (*p* = 0.02 and *p* = 0.009, accordingly). Two-way ANOVA—(F_3, 75_ = 2.89, *p* = 0.041).

### 3.3. PCA Analysis

**Figure 7:**
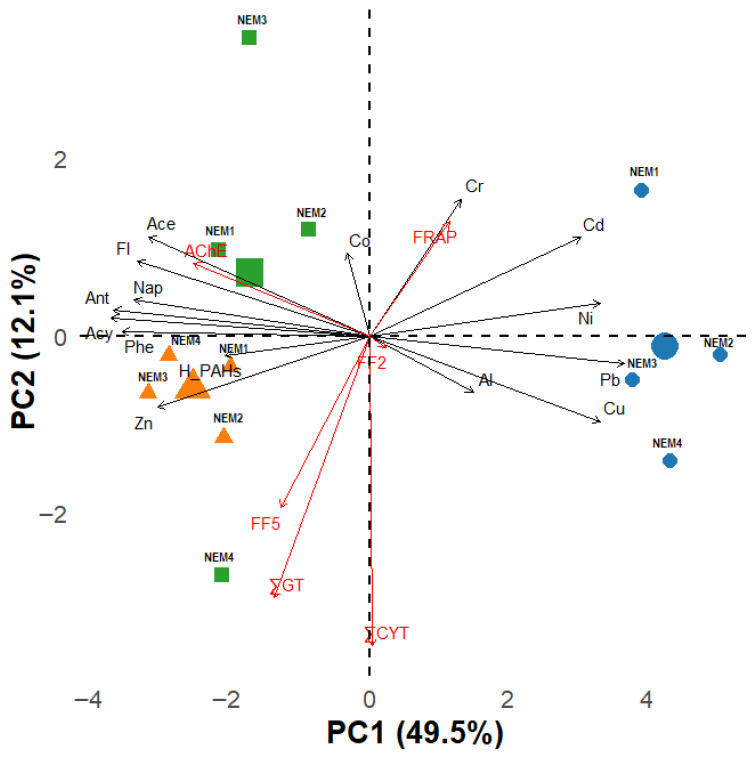
PCA biplot showing relationships between variables (metals and PAHs) and studied biomarkers (∑GT, ∑CYT, AChE, FRAP, FF2, FF5) measured in *Unio pictorum* from four sites along the Nemunas River over three years. Black and red arrows represent measured chemicals and biological response variables, respectively; circles, triangles, and squares correspond to samples from 2020, 2021, and 2022.

The first two principal components, PC1 and PC2, which explained 49.5% and 12.1% of the total variance, respectively, were retained for visualization (Figure 7).

In 2020, the ordination clearly separated stations influenced by metal pollution from other years of the study. The NEM_1 station differed from other downstream stations and was mostly influenced by Cd, Ni, and Cr. Stations downstream were affected by Pb, Cu, and Al. During the second year, all study stations formed a relatively homogeneous cluster. PCA indicates that those results were strongly driven by high-molecular-weight PAHs (H_PAHs) and Zn. In 2021, the ordination exhibited increased dispersion, reflecting greater heterogeneity in environmental conditions and their influence on biomarker responses.

Bioindicators showed a strong positive association between Cr concentrations and FRAP values. Strong loadings were observed between AChE and low-weight PAHs such as Ace, Fl, Nap, Ant, and Acy. The FF5 biomarker shows the appearance of benzo(α)pyrene type metabolites in *U. pictorum*, which is a well-known genotoxin [40]. PCA analysis shows a robust relationship between FF5 and ∑GT.

## 4. Discussion

A three-year study was performed by using classical chemical analysis (determination of PAHs and trace metals in biological material) and a battery of exposure (PAH metabolites) and effect-based biomarkers (AChE activity and the levels of total antioxidant capacity, as well as genotoxicity and cytotoxicity) in native mussels *U. pictorum* from four areas of the Nemunas River to assess the impact of discharged FW after a large-scale fire in a tire warehouse, including potential effects of runoff from a territory affected by atmospheric fire emission. Long-term assessment of the environmental status of the Nemunas River has shown that filter-feeding bioindicators—*U. pictorum*—were under toxic stress.

Right after the fire, observations of air quality, sediments and water in the affected areas revealed elevated levels of pollutants, indicating widespread environmental disturbance resulting from the incident. The Lithuanian Environmental Protection Agency modelled pollutant emissions during the tire fire in Alytus, revealing that pollutants primarily spread northwest and northeast, away from the living districts of Alytus City. The highest air pollutant concentrations were detected within several kilometres’ radius of the fire site from the second to the fourth day of tire burning [41]. In soil from the Alytus tire fire-affected area, particularly Cr, Zn, Ni, Cu, and to a lesser extent Pb, as well as high levels of total PAHs, were detected, with concentrations reaching up to 5872 ng/g dw [1]. Low-molecular-weight PAHs, especially Nap (about 17–83% of the total mass of 16 PAHs), dominated in the immediate vicinity of the fire, whereas more distant areas exhibited higher concentrations of 4-ring compounds like Pyr and Flu. Zinc was the most abundant metal found, with levels reaching 227.5 μg/g [1]. Principal component analysis revealed that areas near the fire experienced the greatest impact from these contaminants. In the area affected by the Alytus tire fire, surface water bodies analysis (2019–2022, at 18 sites) showed that river and pond water corresponded to the class of bad or very bad ecological condition [42]. The study performed by Gefenienė and co-authors presents that in firefighting water used in the Alytus fire(FW), Cu and Pb concentrations were 18 and 286 times higher, respectively, than those found in the Nemunas River. Zinc and manganese recorded the highest levels among the heavy metals detected in the Alytus FW, with concentrations of 580 μg/L and 1213 μg/L, respectively. Higher concentrations of PAH, as well as other compounds commonly associated with tire fires, were found [2]. Additional contamination by PAHs and trace elements, such as As, Zn, Al and Cu, which exceed the threshold levels in clay mineral-rich soils, was also demonstrated after a fire at a tire landfill in the surrounding area of Madrid City (Spain) [43]. Higher PAH levels were also detected in soils and in the crops of the affected site after a fire on an illegal landfill located in Sesena (Toledo, Spain) [44,45]. Chemical analysis of the current study also showed a marked increase in Zn concentrations (Figure 2b), while PCA highlights its strong influence on the results obtained in 2021 (Figure 7). Many of the emitted contaminants are also known to be mutagenic and carcinogenic [8,11,12,43]. A tire wear particle leachate containing a variety of metals and PAH induced acute toxicity in rotifer (*Brachionus plicatilis*) [46].

The chemical analysis of *U. pictorum* collected in 2020 revealed relatively lower concentrations of the analysed substances in their tissues, possibly due to restrictions implemented during the COVID-19 outbreak. PAH concentrations declined during the lockdown in the Elbe River [47]. An increasing number of studies have reported pronounced declines in air and water contamination, as well as changes in the dominant emission sources, during the lockdown period [48,49], as well as a significant increase in contamination levels in the following post-quarantine period when the quarantine restrictions were gradually removed [50,51,52].

In the context of reduced environmental pollution, the consequences of the fire and the spillage of water used to extinguish the fire may not have been as severe as they might have been at other times. However, the effects were still visible and were most pronounced in 2020 at the stations downstream from WTP outlet and the NEM_1 station, where the tissues of the collected mussels contained higher concentrations of trace metals (all excluding Zn and Co). However, in 2021, the results demonstrated differences, with accumulated PAH concentrations being more than five times higher than those estimated a year earlier, and changes in the composition of accumulated metals. Those results were confirmed by PCA analysis (Figure 7).

The toxic impact of PAH-metal mixtures depends on several factors: the specific types of PAHs and trace metals involved, the organism exposed, the concentration ratios of the mixture components, the availability of organic ligands with high affinity for PAHs and metals, and the presence of multiple environmental contaminants [53]. Numerous studies have reported toxic interactions between PAHs and metals that can lead to more-than-additive co-toxicity, particularly through mechanisms involving disrupted cellular transport, impaired detoxification, and redox imbalance [53]. Both chemical groups are known to induce oxidative stress. PAHs can promote the generation of reactive oxygen species (ROS) via biotransformation pathways, while certain metals participate in Fenton-type reactions, which are a significant source of ROS. Their combined effects on redox balance may be synergistic, as they can influence bioaccumulation and detoxification processes. The resulting ROS can damage vital cellular components, including lipids, DNA, and proteins, leading to oxidative stress and cellular dysfunction [54].

Biochemical biomarker, AChE enzyme activity, assessed in haemolymph of *U. pictorum* from station NEM_3 (2020), revealed a decrease in comparison to the levels measured in specimens collected in 2021 and 2022. This may suggest that the organism responds to emerging environmental pollutants, such as naphthalene-type PAH metabolites. It is important to note that this station had the highest amount of these metabolites compared to other stations. Inhibition of AChE activity was measured in fish (*Anabas testudineus*) treated for 72 h with various concentrations of naphthalene [55], in bivalves (*Perna perna* and *Mytilus trossulus*) after exposure to different trace metals [56,57]. Shen and co-authors have reported that the low-molecular-weight PAHs exhibited lower spatial variability between the study sites than the high-molecular-weight PAHs, a pattern attributed to their higher solubility in water and enhanced mobility in the aquatic system [58]. Furthermore, they demonstrated that mussels accumulate substantially lower concentrations of low-molecular-weight PAHs in comparison to high-molecular-weight congeners during the process of filter feeding.

In the present study, the lowest levels of antioxidant capacity using the FRAP method were detected in *U. pictorum* collected at the river station NEM_2 in 2021 (Figure 1). Low FRAP values assessed in 2021 differed (based on post hoc Fisher LSD tests) from the values measured in 2020, and 2022. PCA analysis revealed a strong relation between FRAP and Cr. Across the dataset, a progressive antioxidant response was observed, indicating a potential protective role against oxidative stress and ROS generation induced by metal contamination. FRAP serves as an indicator of molecules’ potential to modulate metal-driven oxidative processes and represents heavy-metal stresses in the investigated *U. pictorum*. Oxidative stress induced by hypoxia in mussel’s hemolymph via FRAP assay was demonstrated [16]. A potential use of the FRAP assay as a biomarker of oxidative stress in mussels (*Mytilus galloprovincialis*) was assessed earlier in studies by Franco-Martínez and co-authors [16]. FRAP with other performed biochemical and histochemical biomarkers (protein carbonyls, lipofuscin, lysosomal stability (N-acetyl-b-hexosaminidase activity, malonaldehyde) of oxidative and general stress demonstrated a gradient of effects in the most impacted organisms found in the upper Tamar estuary [59]. A significant decrease in antioxidant capacity value was reported in blue mussels *M. galloprovincialis* affected by metals and organic pollutants under laboratory conditions [60].

It is known from earlier studies that mussels accumulate PAHs and trace metals from the environment and metabolize these compounds [61]. Our results demonstrate that the highest levels of benzo(a)pyrene-type PAH metabolites were detected in *U. pictorum*, which were collected in the study site NEM_3 in the second year of the study (2021). PCA revealed that the presence of benzo(α)pyrene (FF5-type PAH metabolites) in *U. pictorum* hemolymph is strongly associated with genotoxic alterations in gill cells. The genotoxic and cytotoxic effects of PAHs, particularly benzo(α)pyrene, have been extensively documented in a wide range of taxa, including bivalves [40].

The highest values of genotoxic aberrations were registered in mussels at station NEM_2 during all three-year surveys (4.9‰, 4.76‰, and 5.53‰, respectively), while the highest total cytotoxicity levels at this site were assessed only in 2020—4.4‰ and 2022—4.89‰ (Figure 6). This could be explained by the release of genotoxic and cytotoxic agents into the aquatic environment after the fire, general pollution, and urban run-off from the upstream-located water treatment plants. The significant differences in genotoxicity levels found in 2020 between mussels from the close stations NEM_1 and NEM_2 were possibly caused by discharges of the WTP. The post hoc Fisher LSD test also highlights that the ∑GT values measured at the NEM_2 station were higher than those measured at all other study stations. However, it cannot be stated that FW discharge was the prevailing source of long-term contamination with PAHs. Pyrogenic and petrogenic pollution could be possibly related to municipal and industrial effluents, deposition of solid particles from the air, motorboats, emissions from land sources, exhaust gases from vehicles, and incineration of agricultural waste [25]. The impact of these pollution sources has increased markedly since the removal of COVID-19 restrictions. The impact of PAHs was highlighted by PCA in 2021 and 2022.

In the present study, biochemical and cytogenetic biomarker surveys conducted in 2022 revealed that the levels of the studied parameters were lower in mussels collected from stations located farther from the city of Alytus. This phenomenon could be associated with the processes of sedimentation, as well as with the biological and biochemical degradation of pollutants. However, it should be noted that studies performed a decade ago indicated that some sections of the Nemunas River were much more affected by contamination originating from municipal and industrial effluents than others [25,62].

## 5. Conclusions

In conclusion, it can be stated that *U. pictorum* mussels collected close to the WTP discharger were under toxic pressure. Used bioindicators had accumulated PAHs and trace metals from their environment, with the highest levels of combustion-derived PAH metabolites observed in specimens gathered near the outlet of the WTP. The mussels from the closest Nemunas station, NEM_2, exhibited signs of generally toxic and neurotoxic stress, with high levels of genotoxic and cytotoxic effects. Results indicated that genotoxic agents had likely been released into the aquatic environment post-fire and after the spillage of water used for firefighting. The presence of toxic metals and PAHs from both historical and recent pollution sources poses a serious threat to the aquatic ecosystem, including all organisms. The study found that benzo(a)pyrene-type PAH metabolites were prevalent at the NEM_3 site in 2021, indicating contamination primarily from combustion processes. Total naphthalene-type PAH metabolites, associated with petrogenic sources, were the highest at this station in 2020. The significant levels of PAH metabolites detected in *U. pictorum,* particularly from NEM_3, were in accordance with increased PAH concentrations assessed in mussels, directly linking biological effects with accumulated pollutants. The results emphasize the potential of the studied biomarkers to be used broadly in aquatic studies to support environmental protection efforts, ensuring the maintenance of water quality and security worldwide, across various ecosystems. However, in order to apply these methods more widely, such factors as sampling season, temperature, and bioindicators’ reproductive period must be considered. Furthermore, the current study includes a period of unique changes in human activities in 2020 (reduced industrial activity, decreased transportation, increases in domestic sewage and medical waste, etc.) related to COVID-19 restrictions, which resulted in changes in anthropogenic contamination. They were gradually restored in 2021 and 2022.

The study performed in 2020 highlights the importance of implementing appropriate treatment of firefighting water, as the results demonstrate a negative impact on the studied bivalve mussels. The possibility that other aquatic organisms, including fish and smaller macroinvertebrates, may also have been affected cannot be ruled out. If such water enters the aquatic environment (during the spillages or accidents), long-term monitoring should be mandatory in order to assess the recovery of the affected ecosystem. Such studies provide valuable information for predicting the potential consequences of similar accidents and evaluating environmental damage.

## Figures and Tables

**Table 1:** Description of the study stations, sampling dates, and number of samples collected.

Station Code	Brief Description of the Station	Geographical Coordinates	Sampling Dates/Number of Mussels (n)
**NEM_1** On the figures denoted accordingly NEM_1/2020, 2021, 2022	The station is located upstream of the Alytus WTP “Dzūkijos vandenys”	54°25′48.9″ N 24°03′13.8″ E	2020.06.27, (25) 2021.06.28, (25) 2022.06.28, (25)
**NEM_2** On the figures denoted accordingly NEM_2/2020, 2021, 2022	The station is situated about 2.5 km downstream from the outlet of the Alytus City WTP and NEM_1 station	54°26′58.3″ N 24°03′56.1″ E	2020.06.27, (25) 2021.06.28, (25) 2022.06.28, (25)
**NEM_3** On the figures denoted accordingly NEM_3/2020, 2021, 2022	The station is located on the territory of Nemunas Loop regional park, about 38 km downstream from the outlet and the NEM_1 station	54°34′01.9″ N 23°58′01.2″ E	2020.06.28, (25) 2021.06.29, (25) 2022.06.29, (25)
**NEM_4** On the figures denoted accordingly NEM_4/2020, 2021, 2022	The station is located about 66–68 km downstream from the Alytus City WTP outlet and the NEM_1 station	54°39′05.8″ N 24°04′53.2″ E	2020.06.27, (25) 2021.06.28, (25) 2022.06.28, (25)

## Data Availability

The original contributions presented in this study are included in the article. Further inquiries can be directed to the corresponding authors.
